# Enhanced insular/prefrontal connectivity when resisting from emotional distraction during visual search

**DOI:** 10.1007/s00429-019-01873-1

**Published:** 2019-05-21

**Authors:** Tiziana Pedale, Emiliano Macaluso, Valerio Santangelo

**Affiliations:** 1 Department of Psychology, Sapienza University of Rome, Via dei Marsi, 78, 00158 Rome, Italy; 2 Neuroimaging Laboratory, IRCCS Santa Lucia Foundation, Via Ardeatina, 306, 00179 Rome, Italy; 3 Umeå Center for Functional Brain Imaging (UFBI), Department of Integrative Medical Biology, Umeå University, 901 87 Umeå, Sweden; 4 ImpAct Team, Lyon Neuroscience Research Center, 16, av. du Doyen Lépine, 69676 Bron Cedex, France; 5 Department of Philosophy, Social Sciences and Education, University of Perugia, Piazza G. Ermini, 1, 06123 Perugia, Italy

**Keywords:** Emotions, Visual search, Task relevance, Everyday life scene, Functional connectivity, fMRI

## Abstract

Previous literature demonstrated that the processing of emotional stimuli can interfere with goal-directed behavior. This has been shown primarily in the context of working memory tasks, but “emotional distraction” may affect also other processes, such as the orienting of visuo-spatial attention. During fMRI, we presented human subjects with emotional stimuli embedded within complex everyday life visual scenes. Emotional stimuli could be either the current target to be searched for or task-irrelevant distractors. Behavioral and eye-movement data revealed faster detection of emotional than neutral targets. Emotional distractors were found to be fixated later and for a shorter duration than emotional targets, suggesting efficient top-down control in avoiding emotional distraction. The fMRI data demonstrated that negative (but not positive) stimuli were mandatorily processed by limbic/para-limbic regions (namely, the right amygdala and the left insula), irrespective of current task relevance: that is, these regions activated for both emotional targets and distractors. However, analyses of inter-regional connectivity revealed a functional coupling between the left insula and the right prefrontal cortex that increased specifically during search in the presence of emotional distractors. This indicates that increased functional coupling between affective limbic/para-limbic regions and control regions in the frontal cortex can attenuate emotional distraction, permitting the allocation of spatial attentional resources toward task-relevant neutral targets in the presence of distracting emotional signals.

**Electronic supplementary material:**

The online version of this article (10.1007/s00429-019-01873-1) contains supplementary material, which is available to authorized users.

## Introduction

In everyday life, distraction can easily interfere with goal-directed behavior, limiting humans’ ability to stay focused on task-relevant information (Eltiti et al. 2005). Previous literature demonstrated that emotional events (mainly negative) have a privileged access to visual awareness because they tend to capture attention and processing resources in a bottom-up fashion (Vuilleumier 2005; Yiend 2010). For the same reason, negative stimuli have been shown to be particularly successful in distracting people from their current goal (Anticevic et al. 2010; Dolcos et al. 2006; Dolcos and McCarthy 2006; Iordan and Dolcos 2015; Wessa et al. 2013). Several studies reported evidence that negative stimuli are hard to be ignored, even when the emotional valence of the stimulus is entirely irrelevant to the current task (Fenker et al. 2010; Huang et al. 2011; Pessoa et al. 2002; Vuilleumier et al. 2001; Ziaei et al. 2014, 2018).

The neural mechanisms that allow for a prioritized processing of task-relevant negative information are thought to involve direct subcortical pathways that reach the amygdala (for reviews, see Pessoa and Adolphs 2010; Vuilleumier 2005) and the insula (Menon and Uddin 2010; Uddin 2015; Zaki et al. 2012). While the role of the amygdala in emotional processing has been suggested since a long time (MacLean 1952; see also Pessoa 2008), recent models emphasize the crucial role played by the insula as an emotional “hub” (Menon and Uddin 2010; Uddin 2015; Wessa et al. 2013; Zaki et al. 2012). This para-limbic region has been demonstrated to derive information about bodily states, and, subsequently, to play a crucial role in the experience of emotions (Critchley and Harrison 2013; Zaki et al. 2012). The insula would detect behaviorally relevant stimuli and coordinate high-level neural resources through extensive anatomical and functional connections with the rest of the brain (Menon and Uddin 2010; Uddin 2015). This region has been shown to have intrinsic functional connectivity with large-scale brain networks such as the dorsal frontoparietal network and the default mode network (e.g., Seeley et al. 2007; Sridharan et al. 2008; Uddin et al. 2011). For these reasons, the insula has been recently described as the core structure of the so-called “saliency network” (Uddin 2015), a brain system devoted to prioritize processing of potentially relevant information (Desimone and Duncan 1995; Itti et al. 1998), with implications for the allocation of spatial attention (e.g., Gottlieb et al. 1998; Nardo et al. 2014) and working memory (WM) encoding (e.g., Fine and Minnery 2009; Melcher and Piazza 2011; Pedale and Santangelo 2015; Santangelo and Macaluso 2013; Santangelo et al. 2015; see, for a review, Santangelo 2015). Overall, both limbic and para-limbic regions (namely the amygdala and the insula) are thought to automatically activate in the presence of negative stimuli and to modulate the activity of key regions involved in sensory processes (e.g., the primary visual cortex; Vuilleumier 2005) and high-level post-perceptual processes, such as top-down attentional control (e.g., the dorsolateral prefrontal cortex; Uddin 2015), short- and long-term memories (e.g., the medial temporal lobe and the hippocampus; Dolcos et al. 2004), and decision-making (e.g., the orbital frontal cortex; Bechara et al. 2000).

The impact of “emotional distraction” on goal-directed behavior has been primarily studied in the context of WM tasks, for example, by presenting task-irrelevant negative stimuli while the participants have to maintain previously encoded information (Anticevic et al. 2010; Dolcos et al. 2006; Dolcos and McCarthy 2006; Iordan and Dolcos 2015). Using this design, Dolcos and McCarthy (2006) reported that the presentation of negative stimuli during the WM maintenance phase evoked increased activity in emotional-related areas, namely the amygdala and the ventrolateral prefrontal cortex. Concurrently, the authors observed a decrement of activation in working memory-related areas, such as the dorsolateral prefrontal cortex and the lateral parietal cortex. The latter imaging effect correlated with a concurrent behavioral decrement of WM performance. In the same year, Dolcos and colleagues (2006) also reported evidence that emotional-related areas, such as the amygdala, showed increased functional connectivity with the inferior frontal cortex—a well-known area involved with general inhibitory processes (e.g., Aron et al. 2004)—when negative distractors were presented during WM maintenance. These findings highlight a tight interplay between ventral “affective” and dorsal “control” regions that shows enhanced coupling to cope with emotional distraction in the context of WM tasks (see, for a review, Iordan et al. 2013).

However, in these previous WM studies, neutral stimuli and emotional distractors were temporally separated, with emotional interference arising in the absence of any simultaneous stimulation. On the contrary, in everyday life, our sensory experience is characterized by a multitude of concurrent stimuli competing among them to access our awareness (Bundesen et al. 2011). Emotionally salient stimuli are thought to have a high probability of winning the competition, affecting the distribution of our attentional resources (Vuilleumier 2005; Yiend 2010). In this sense, visual search tasks could offer an optimal scenario to understand the neural systems that are responsible for the facing of “emotional distraction” on goal-directed behavior during the deployment of visual attention resources. Visual search is an attention task involving an active scan of the environment for a specific target among a number of different distractors. During a visual search task, neutral and emotional objects could be simultaneously presented allowing to test for the efficacy of emotional stimuli in promoting visual attention selection when they are the target to be searched for, or in negatively affecting the capacity to pay attention to other (emotionally neutral) elements when they are task irrelevant (i.e., emotional distraction).

Previous behavioral studies showed that emotional distraction plays a detrimental role on visual search performance (Anderson et al. 2011; Fenker et al. 2010; Hodsoll et al. 2011; Huang et al. 2011). However, as far as we know, no studies have been conducted to investigate the specific neural correlates involved with the avoidance of task-irrelevant emotional stimuli during visual search. Moreover, only few studies directly compared searching for emotional vs. non-emotional targets (i.e., with the emotional item playing a distracting role). These studies reported contrasting results, and none of these investigated the neural correlates involved with these processes. Hodsoll and colleagues (2011) reported a behavioral study in which participants were asked to search for a female target face among male distracting faces (or vice versa) and judge whether the target face was tilted to the left or to the right. When one of the distractor faces had an emotional expression, the orientation discrimination of the target face was impaired. This suggests that task-irrelevant emotional stimuli can capture attention resources, with a consequent detriment on search performance. Other studies showed, however, opposite findings. Hunt and colleagues (2007) asked participants to make a speeded saccade toward a predefined target among distractors. The valence (happy or angry) and orientation (upright or inverted) of the target and distractors, both consisting in “emoticon” faces, varied. The authors reported that task-irrelevant emotional faces captured oculomotor behavior, thus impairing search of the current target. However, this happened only when the current target was defined by an emotional expression. By contrast, when the participants were asked to search for a neutral feature, such as an upright face among inverted distractors, task-irrelevant emotional faces failed to capture the overt orienting of attention. The authors interpreted these results as an evidence that searching for neutral stimuli in the presence of emotional distractors depends on top-down attention control (e.g., Pessoa et al. 2002), and on the specific task-set related to the current target definition.

Overall, this behavioral literature indicates that under some circumstances emotional stimuli have privileged access to attentional and perceptual processes, while in other conditions efficient top-down regulation can prevent distraction/interference. Here, we conducted an eye tracking–fMRI experiment aimed at investigating—both at behavioral and neuro-physiological level—the interplay between emotional capture and emotional distraction (derived from either negative or positive stimuli), that is, the impact of task-relevant vs. -irrelevant emotional objects in biasing spatial attention selection. With respect to the previous literature, employing very simple and repetitive stimuli (e.g., words: Bradley and Lang 1999; faces: Lundqvist et al. 1998; single visual objects: IAPS, Lang et al. 1999), here we used complex everyday life scenes. Complex scenes involve a large number of discrete elements, thus enhancing stimulus competition and the need of attentional selection (e.g., Henderson 2003; see also Desimone and Duncan 1995). We hypothesized that the interplay between affective and control regions affects the allocation of spatial attention when searching through visual scenes that include emotional stimuli. Moreover, we asked whether any such mechanism of attention control would engage also when distraction derives from positive stimuli or it is rather selective for coping with negative-driven emotional distraction.

During fMRI scanning, we presented participants with pictures depicting everyday scenes. These included an emotional object (either negative or positive) that in half of the trials corresponded to the to-be-searched and judged target. When emotional objects were task irrelevant, subjects were asked instead to search for an emotionally neutral object in the scene. Additionally, we added a baseline condition, consisting of scenes not including any emotional object, which enabled us to measure the behavioral performance and neural correlates of searching for a neutral object in the absence of emotional distraction. At a behavioral level (Behavioral Hypothesis, Beh H 1), we expected a “search benefit” for task-relevant emotional targets compared to neutral targets (Vuilleumier 2005; Yiend 2010). Following the literature on emotional distraction that mainly investigated the effect of “negative” distracting stimuli (Anderson et al. 2011; Anticevic et al. 2010; Dolcos et al. 2006; Dolcos and McCarthy 2006; Hodsoll et al. 2011; Iordan and Dolcos 2015; Wessa et al. 2013; Ziaei et al. 2014, 2018), we also predicted (Beh H 2) a “search cost” when the participants had to find neutral targets in scenes including a task-irrelevant negative distractor compared to scenes without emotional distractor.

Furthermore, we collected eye-movement data, which allowed us to assess the exploration of the scenes depending on the task relevance/irrelevance of the emotional stimuli. Here, we expected (Eye Movement Hypothesis, EM H 1) that task-relevant emotional objects would lead to faster fixations compared to neutral targets. Additionally (EM H 2), if emotional objects were automatically processed we would expect to find evidence of equally fast fixations, irrespectively of their task relevance. By contrast (EM H 3), if top-down control was efficient in avoiding emotional distraction we would expect a reduction of attentional capturing by task-irrelevant vs. task-relevant emotional stimuli (Huang et al. 2011; Hunt et al. 2007).

At a neuroimaging level (fMRI Hypothesis, fMRI H 1), we expected that searching for emotional objects would reflect in the activation of limbic (i.e., the amygdala; Vuilleumier et al. 2001) and para-limbic (i.e., the insular cortex; Uddin 2014) areas. Moreover (fMRI H 2), we expected that coping with emotional distraction when searching for a neutral target would result in an increased activation of brain regions involved in top-down attention control, such as the dorsal frontoparietal network to preserve goal-directed behavior (Corbetta et al. 2008; Corbetta and Shulman 2002; see also Iordan et al. 2013; Wessa et al. 2013; Ziaei et al. 2014). While the previous literature on emotional distraction (Anticevic et al. 2010; Dolcos et al. 2006; Dolcos and McCarthy 2006; Iordan and Dolcos 2015) mainly focused on the interference driven by negative distractors, in the present study we also aimed at investigating whether positive stimuli would produce a similar interference, and would engage the same coping mechanism at the neural level. Specifically, we expected an increased activation of regions related to voluntary eye-movement control—such as the frontal eye field (FEF; Mohanty et al. 2009; Tseng et al. 2014)—during the avoidance of both negative and positive emotional distractors. Further (fMRI H 3), on the basis of previous literature suggesting automatic processing of negative stimuli by limbic/para-limbic areas (i.e., the amygdala and the insular cortex; e.g., Phelps 2006; Uddin et al. 2014; Vuilleumier et al. 2001), we tested for higher activation of those areas specifically involved with the processing of negative stimuli, irrespective of their task relevance, also with the help of a localizer task for emotional-related processing areas. Finally (fMRI H 4), following the hypothesis that affective regions modulate the activity of the frontoparietal control regions during negative emotional distraction (Dolcos and McCarthy 2006), we expected an increased functional connectivity between limbic/para-limbic areas (mainly responding to negative stimuli; i.e., the amygdala and the insular cortex; e.g., Phelps 2006; Uddin et al. 2014) and the dorsal frontoparietal control network when subjects searched for a neutral target-object in the presence of a negative emotional distractor. This would be consistent with the notion that the interplay between affective and attention control regions can mitigate the impact of emotional distraction on the allocation of spatial processing resources.

## Materials and methods

### Participants

Twenty-five healthy volunteers took part in the experiment. Three participants were excluded from data analysis because of within-fMRI-run head movements larger than 3 mm or 3°, leaving 22 participants for the final analyses (10 males; mean age 23.6 years; range 19–30 years). All participants gave written consent to the study, which was approved by the independent Ethics Committee of the Santa Lucia Foundation. All procedures were in accordance with the principles of the 1964 Helsinki declaration.

### Stimuli and task

The set of stimuli included 150 pictures depicting scenes of everyday life. These pictures were collected on the World Wide Web and included both internal (e.g., kitchens, living rooms, bedrooms, etc.) and external scenes (e.g., roads, buildings, natural landscapes, etc.), but no single-object photo. No people were represented in any scene. Pictures were displayed at 18 × 12° of visual angle (resolution in pixels, 680 × 448). 120 of the 150 pictures were digitally modified by means of CorelDraw Graphics Suite v. 12 to include negative or positive emotionally arousing objects, also collected on the World Wide Web (i.e., emotional scenes). 60 pictures included a negative emotional stimulus (e.g., a spider, a snake, etc.), while the other 60 pictures included a positive emotional stimulus (e.g., a cake, a puppy, etc.; see, for a similar approach, Buttafuoco et al. 2018). The remaining 30 pictures did not include any emotional stimulus (neutral scenes).

To ensure that the inclusion of the emotional object affected the emotional impact of the scenes, we asked a group of 50 independent observers not taking part in the main experiment (18 males; mean age = 30.0 years, range 21–61 years) to rate the emotional valence (9-point scale: 1 = totally negative, 9 = totally positive) and the emotional arousal (9-point scale: 1 = totally calm, 9 = totally excited) of the scenes by means of an online survey. Crucially, the comparison between emotional and neutral scenes revealed that the emotional scenes significantly influenced emotional valence (mean ± standard error for scenes with negative object vs. neutral scenes: 2.64 ± 0.09 vs. 5.55 ± 0.15, *t*(88) = − 17.4, *p* < 0.001; scenes with positive object vs. neutral scenes: 6.22 ± 0.10 vs. 5.55 ± 0.15; *t*(88) = 4, *p* < 0.001), and emotional arousal (scenes with negative object vs. neutral scenes: 6.03 ± 0.11 vs. 3.33 ± 0.07, *t*(88) = 16.9, *p* < 0.001; scenes with positive object vs. neutral scenes: 3.76 ± 0.06 vs. 3.33 ± 0.07; *t*(88) = 4.2, *p* < 0.001). Overall, these data provide clear evidence that the inclusion of the emotional object (either negative or positive) made the entire scenes perceived and evaluated as more emotionally loaded than neutral scenes.^1^

**Fig. 1:**
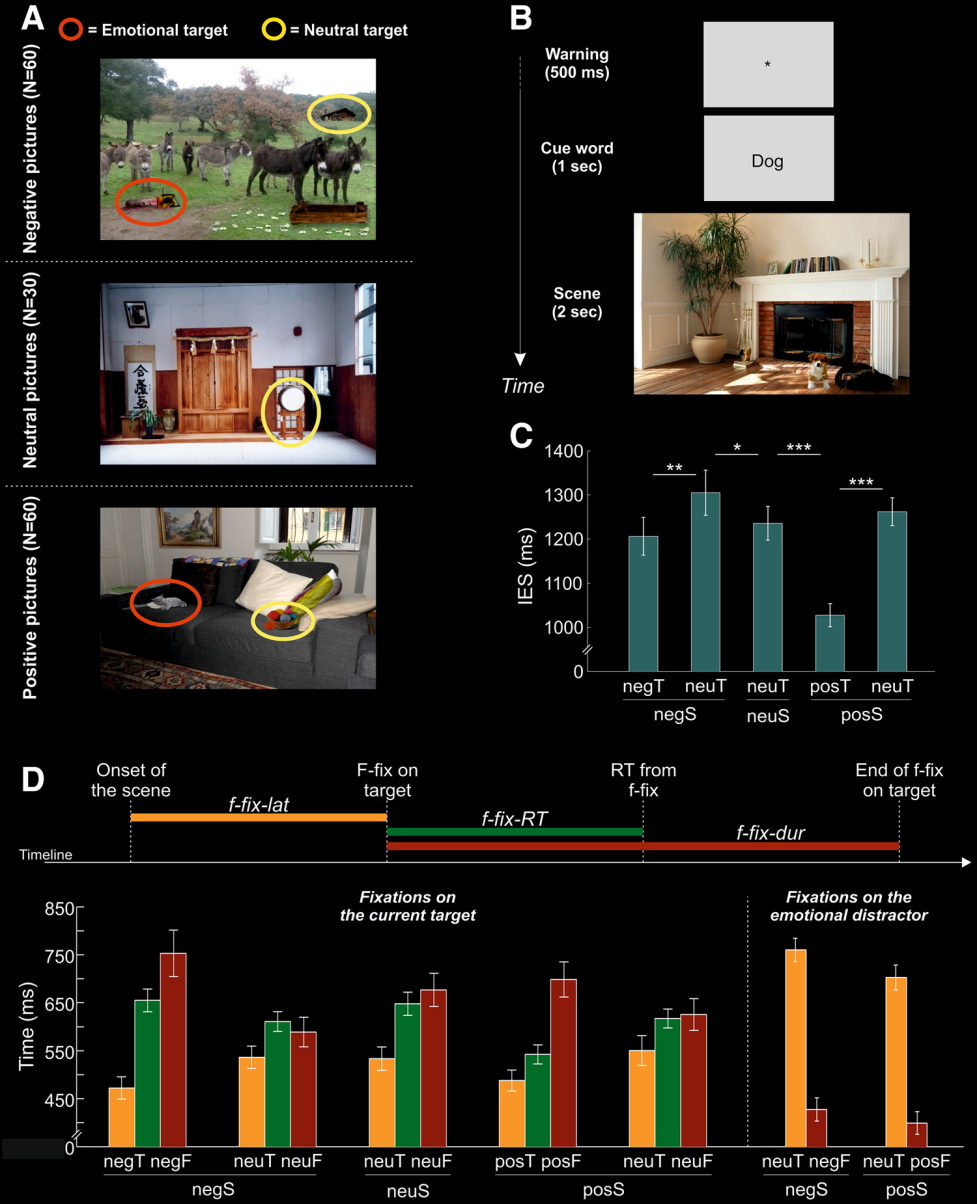
**a** Examples of negative, neutral and positive scenes including emotional target-objects (red circles in negative and positive scenes) and neutral target-objects (yellow circles in negative, neutral and positive scenes). Circles are not displayed during the experiment. **b** Diagram showing the sequence of events during one trial. The trial began with a warning signal for 500 ms. The cue word was then presented for 1 s. This defined the to-be-searched target in the following scene, which was presented for 2 s. Within this interval, participants had to search for and discriminate the position (left vs. right) on the scene of the pre-cued target-object by pressing one of two response buttons. After a variable ITI ranging from 8.5 to 10.5 s a new trial began. **c** Behavioral results. Mean inverse efficiency scores (IES) ± standard error of the means for the five conditions: negS_negT, negS_neuT, neuS_neuT, posS_posT, posS_neuT. Asterisks indicate statistically significant differences between conditions: *p < 0.05; **p < 0.01, ***p < 0.001. **d** Fixations patterns. Top panel: timeline indicating the meaning of the different fixation indexes, i.e., the latency of the fixation on the target-object (f-fix-lat, yellow bar), the RT measured from the onset of the first fixation on target (f-fix-RT, green bar); and the duration of this first fixation (f-fix-dur, red bar). Bottom panel: Mean ± standard error of f-fix-lat, f-fix-RT, and f-fix-dur related to fixations of the current target-object in the main conditions (negS_negT_negF, negS_neuT_neuF, neuS_neuT_neuF, posS_posT_posF, posS_neuT_neuF) or to fixations of the emotional object when it was not the to-be-searched target in the negS_neuT_negF and posS_neuT_posF conditions

During fMRI scanning, participants were asked to localize and report the position (left vs. right hemifield) of the target-object, corresponding to a cue word presented at the beginning of each trial. For each emotional scene, we designated as the to-be-searched object either the emotional object or another, emotionally neutral object presented in the scene. For the neutral scenes, we designated only one object as the search target. For each emotional scene, the identity of the search target (emotional or neutral object) was counterbalanced across participants. This generated five different search conditions (see Fig. 1a):

- scenes including a to-be-searched negative target (negS_negT; i.e., negative scene, negative target; 30 pictures);
- scenes including a negative emotional object wherein the to-be-searched target was a neutral object (negS_neuT; i.e., negative scene, neutral target; 30 pictures);
- scenes including a to-be-searched positive target (posS_posT; i.e., positive scene, positive target; 30 pictures);
- scenes including a positive emotional object wherein the to-be-searched target was a neutral object (posS_neuT; i.e., positive scene, neutral target; 30 pictures);
- scenes not including any emotional stimulus with a neutral to-be-searched target (neuS_neuT; i.e., neutral scene, neutral target; 30 pictures), used as a baseline condition.

Targets were located equiprobably in the left or the right hemifield. Several evaluations of the pictures were performed to control for possible differences between the experimental conditions. First, we made sure that the size of the target-objects did not differ significantly between the five experimental conditions, as revealed by a one-way ANOVA, [*F*(4, 116) < 1; n.s.]. Then, we checked for the eccentricity of the target-objects across the five conditions. Separately for each picture, we computed the center of mass of the target-object as the average of the horizontal and vertical coordinates of each pixel belonging to the target-object. We extracted the horizontal eccentricity considering the horizontal position of the center of mass and converted this to degrees of visual angle. The absolute horizontal eccentricity values did not differed across the five conditions: [*F*(4, 116) < 1; n.s.]. Finally, we measured the visual saliency of target-objects using the Saliency Toolbox 2.2 (http://www.saliencytoolbox.net/). This created a saliency map for each scene using local discontinuities in line orientation, intensity contrast, and color opponency (Itti et al. 1998), allowing us to evaluate the saliency associated to the designed target-objects. Saliency values did not differed across the five experimental conditions: [*F*(4, 116) < 1; n.s.]. These quantitative measures helped us to rule out several possible confounding factors in the interpretation of our results.

The participants’ task was to find the target-object and to report whether this was located on the left or the right side of the picture. The presentation of the stimuli and the collection of responses were accomplished through MatLab 7.1 (The MathWorks Inc., Natick, MA), using Cogent 2000 Toolbox (Wellcome laboratory of Neurobiology, University College London). The sequence of events is illustrated in Fig. 1b. Each trial started with the presentation of a warning signal for 0.5 s. Immediately after, a “cue word” defining the to-be-searched target was presented on a gray background for 1 s. This was followed by the presentation of the visual scene. Participants were asked to search for the cued object and to press one of two response buttons as quickly and as accurately as possible, depending on the location of the target-object in the picture (left vs. right visual hemifield). Participants had a maximum time of 2 s to give an answer, and after that the scene disappeared. To optimize the ability to isolate the hemodynamic response associated with each single scene presentation we used a long variable inter-trial interval ranging from 8.5 to 10.5 s (mean of 9.5 s), uniformly distributed (see, e.g., Dale 1999), consisting in a gray background. Participants underwent three fMRI-runs (lasting approximately 11 min each), including 50 trials each. The order of trials within and across runs was randomized with a constraint: each run included ten trials for each of the five conditions.

### Eye movements and fixation indexes

Together with the behavioral data, we also acquired the subjects’ gaze-position during fMRI. This was done to provide us with additional information about the impact of the emotional stimuli on the overt exploration of the visual scene, as a function of task relevance (see, for a similar approach, Santangelo and Macaluso 2013; Santangelo et al. 2015). The eye-movement data were recorded with an ASL eye-tracking system, adapted for use in the scanner (Applied Science Laboratories, Bedford, MA; Model 504, sampling rate 60 Hz). Using the MTtools (http://www.brainreality.eu/mt_tools/), we computed fixation positions for each picture and for each participant considering a time window of 2 s, corresponding to the duration of the scene presentation, starting from the picture onset. We then computed for each picture a target-map. This procedure consisted in manually drawing (using CorelDraw Graphics Suite v. 12) the target-object within each scene and then copy and paste this object on a picture with the same resolution of the initial scene (680 × 448 pixels) consisting on a gray background uniformly distributed. The object was pasted at the same location as the original scene. Hence, for each target we obtained a 680 × 448 matrix with values set equal to 1 at the coordinates of the target-object and zeros everywhere else. Finally, this binary map was smoothed with a Gaussian filter (FWHM = 1°) to account for eye-tracking noise. For the emotional scenes, we built both a target-map related to the emotional object (i.e., negS_negT and posS_posT conditions) and a target-map related to the neutral object (i.e., negS_neuT and posS_neuT conditions); while for the neutral scenes, we built a target-map related to the only neutral object used as target (i.e., neuS_neuT).

These target-maps were used to compute three different fixation indexes: (1) the latency of the first fixation on the target-map (f-fix-lat), indicating the strength of attentional grabbing of the current target; (2) the time interval between the onset of the first fixation on the target and the response button press (f-fix-RT), highlighting the time needed to decide whether the stimulus was a target, having accounted for the initial capture of spatial attention, i.e., the “f-fix-lat” index; and (3) the duration of the first fixation on the target-map (f-fix-dur), indicating the length of perceptual processing devoted to that stimulus, irrespective of the response time, cf. “f-fix-RT”. Figure 1d (top panel) shows a schematic depiction of the three indexes in relation to each other (see also the Online Resource, where we confirmed our main results using a forth fixation-index, namely the fixation probability of the target-map).

We used these fixation indexes to address several hypotheses about the processing of target and non-target emotional stimuli. If emotional targets were attentional “grabbing” (EM H 1) we would expect a bottom-up effect on gaze direction evidenced by: faster first fixation latencies (f-fix-lat), shorter intervals between target fixation and response button press (f-fix-RT), and longer fixation durations (f-fix-dur) following the presentation of emotional compared to neutral targets (cf. Calvo and Lang 2004). To test this hypothesis, we computed f-fix-lat, f-fix-RT, and f-fix-dur related to the object that was defined as target within each scene (see Fig. 1d, bottom panel): (1) task-relevant emotional targets in negative scenes (negS_negT_negF, meaning: negative scene, negS, negative target, negT, analysis related to the negative target, negF); (2) neutral targets in scenes including a negative distractor (negS_neuT_neuF, i.e., negative scene, negS, neutral target, neuT, analysis related to the neutral target, neuF); (3) neutral targets in scenes that not included any emotional distractor (neuS_neuT_neuF, i.e., neutral scene, neuS, neutral target, neuT, analysis related to the neutral target, neuF); (4) task-relevant emotional targets in positive scenes (posS_posT_posF, i.e., positive scene, posS, positive target, posT, analysis related to the positive target, posF); (5) neutral targets in scenes including a positive distractor (posS_neuT_neuF, i.e., positive scene, posS, neutral target, neuT, analysis related to the neutral target, neuF).

Additionally, we characterized fixation patterns also for those conditions in which the emotional object was task irrelevant, that is, a distractor. If emotional objects were automatically processed when task irrelevant (EM H 2), we would expect to find evidence of fast gaze orienting also in these conditions. By contrast, if top-down control was efficient in avoiding emotional distraction (EM H 3) we would expect a reduction of attentional capture by emotional distractors. To check for this, we directly compared fixation indexes related to the emotional object in conditions of task relevance (conditions 1 and 4 above, negS_negT_negF and posS_posT_posF) vs. conditions of task irrelevance, that is: (1) negS_neuT_negF, i.e., negative scene, negS, neutral target, neuT, analysis related to the negative emotional distractor, negF; (2) posS_neuT_posF, i.e., positive scene, posS, neutral target, neuT, analysis related to the positive emotional distractor, posF.^2^

### Magnetic resonance imaging

A Siemens Allegra (Siemens Medical Systems, Erlangen, Germany) operating at 3T and equipped for echo-planar imaging (EPI) was used to acquire the functional magnetic resonance images. A quadrature volume head coil was used for radio frequency transmission and reception. Head movements were minimized by mild restraint and cushioning. Thirty-two slices of functional MR images were acquired using blood oxygenation level-dependent imaging (3 × 3 mm^2^ in plane resolution, 2.5 mm thick, 50% distance factor, repetition time = 2.08 s, time echo = 30 ms), covering the entirety of the cortex.

### fMRI data analysis

We used SPM8 (Wellcome Department of Cognitive Neurology) implemented in MATLAB 7.4 (The MathWorks Inc., Natick, MA) for data pre-processing and statistical analyses. Each participant underwent three fMRI-runs, each comprising 320 volumes. After having discarded the first four volumes of each run, all images were corrected for head movements. Slice-acquisition delays were corrected using the middle slice as a reference. All images were normalized to the standard SPM8 EPI template, resampled to 2 mm isotropic voxel size, and spatially smoothed using an isotropic Gaussian kernel of 8 mm FWHM. Time series at each voxel for each participant were high-pass filtered at 220 s and pre-whitened by means of autoregressive model AR(1).

Statistical inference was based on a random-effects approach, which is comprised of two steps: first-level multiple regression models estimating contrasts of interest for each subject, followed by the second-level analyses for statistical inference at the group level. The main aim of the study was to assess the impact of emotional objects in capturing attention/perceptual resources depending on their current task relevancy: that is, when the emotional object was the search target vs. a distracting object. Accordingly, the first-level model considered as events-of-interest the five main conditions: negS_negT, negS_neuT, neuS_neuT, posS_posT, posS_neuT. All the erroneous and missing trials (i.e., trials in which participants did not correctly localize the left vs. right position of the current target or failed to do so within the time window of 2 s: overall 11.2% of trials) were modelled as a separate event type that was not considered in the group analyses. The events were modelled as miniblocks, time locked at the onset of the pictures with a duration of 2 s, i.e., the time of scene presentation. All predictors were convolved with the SPM8 hemodynamic response function, and the parameters of head movements were included as covariates of no interest. For each subject, linear contrasts were used to average the parameter estimates associated with each of the five conditions of interests, across the three fMRI-runs.

For the group-level analysis, we carried out a within-subject ANOVA that modelled the five relevant event types: negS_negT, negS_neuT, neuS_neuT, posS_posT, posS_neuT. Correction for nonsphericity (Friston et al. 2002) was used to account for possible differences in error variance across conditions, arising—for example—because of the different number of trials in the five conditions of interest and/or any non-independent error terms for the repeated measures.

#### Target-related fMRI analysis

To address our main question about the role of task relevance on the processing of emotionally arousing stimuli, we considered the conditions when the emotional object was either the search target or a task-irrelevant distractor, irrespective of the emotional valence. We used the contrast “target vs. distractor” emotional objects [(negS_negT + posS_posT) > (negS_neuT + posS_neuT)] to identify the activity associated with the effect of searching for a task-relevant emotional stimulus (see fMRI H 1). The reverse contrast, comparing “distractor vs. target” emotional objects [i.e., (negS_neuT + posS_neuT) > (negS_negT + posS_posT)], was used instead to highlight top-down volitional control involved with avoiding non-target (distracting) emotional stimuli, thus preserving goal-directed attention toward the neutral target (see fMRI H 2). Moreover, we checked whether top-down control involved with distraction avoidance was selectively deployed for negative or positive distractors [i.e., (negS_neuT—negS_negT) > (posS_neuT—posS_posT) and (posS_neuT—posS_posT) > (negS_neuT—negS_negT)]. Importantly, it should be noticed that the four conditions considered in these contrasts included scenes containing one emotional object (either the target or the distractor), thus removing any overall effect of processing emotional stimuli. The statistical threshold was set to *p* = 0.05, FWE corrected at the voxel level, considering the whole brain as the volume of interest.

#### Emotional-related fMRI analysis

Since the contrast “target vs. distractor” emotional object described above in the target-related fMRI analysis (negative and positive scenes collapsed together) failed to reveal any significant effect at the whole brain level (cf. “Target-related fMRI analysis”, below), we asked whether any effect of emotional target and/or emotional distractor was specific for the negative stimuli, irrespective of their task relevance (Fenker et al. 2010; Huang et al. 2011; see fMRI H 3). First of all, we identified regions involved in the processing of negative stimuli by comparing conditions including a negative stimulus (negS) vs. all the other conditions: i.e., [(negS_negT + negS_neuT) > (neuS_neuT + posS_posT + posS_neuT)], weighted as: [(1/2 + 1/2) > (1/3 + 1/3 + 1/3)]. The choice to compare negative conditions versus the average of both positive and neutral conditions was motivated by the fact that “negative minus positive” and “negative minus neutral” showed very similar patterns of activation (see Figure S2 in the Online Resource). Moreover, we also performed the opposite comparison, contrasting positive scenes vs. all the other conditions: i.e., [(posS_posT + posS_neuT) > (neuS_neuT + negS_negT + negS_neuT)]. The statistical threshold was set to *p* = 0.05, FWE corrected at the voxel level, considering the whole brain as the volume of interest. In addition, we considered an independent dataset (cf. “Localizer task”, below) that allowed us to focus on limbic and para-limbic areas traditionally involved in the processing of negative stimuli (amygdala and insular cortex, see Phan et al. 2004; Seara-Cardoso et al. 2015). Accordingly, corrected *p* values were also assigned considering the limbic regions identified in the localizer scan as a reduced volume of interest (small volume correction, SVC; Worsley et al. 1996). For this, we used spheres of 8 mm of radius that matched the FWHM of the smoothing filter. Spheres were centered in the left and the right amygdala (cf. Table 2).

Finally, all the regions involved in the processing of negative vs. positive or neutral conditions were combined together in a volume of interest (VOI) using MarsBar 0.42 (“MARSeille Boîte A Région d’Intérêt” SPM toolbox). Within this VOI, we tested the contrasts “distractor vs. target” and “target vs. distractor” emotional objects, now considering only scenes including negative stimuli: (negS_neuT) > (negS_negT) and (negS_negT) > (negS_neuT), respectively; *p*-*FWE*-*corr* = 0.05, at the voxel level. This latter analysis allowed us to check whether the brain activity observed during the processing of negative scenes was modulated by the task relevance of negative objects.

#### Inter-regional connectivity of the affective regions

Together with the intra-regional analyses described above, we performed analyses of inter-regional connectivity to address the hypothesis that coping with emotional distraction may involve changes of connectivity between limbic/para-limbic regions and cortical regions involved in attention control (cf. Dolcos et al. 2006; Iordan et al. 2013; Uddin 2015; see fMRI H 4). We used analyses of inter-regional connectivity [psychophysiological interactions (PPIs)] (Friston 2004) implemented with the “Generalized Form of Context-Dependent Psychophysiological Interactions” SPM toolbox (McLaren et al. 2012). At the subject level, each PPI analysis included five regressors corresponding to the psychological variables of interest (i.e., negS_negT, negS_neuT, neuS_neuT, posS_posT, posS_neuT, as in the main analysis), the time course of the seed area (i.e., the physiological variable highlighting the activity of either the left insula and the right amygdala; cf. Table 2), and the critical cross-products (i.e., the psychophysiological interaction term) between the five psychological variables and time course of each seed area. The head motion realignment parameters were included as covariates of no interest. For each of the two seed areas, the parameter estimates of the five PPI regressors entered a within-subject ANOVA for statistical inference at the group level. For each ANOVA, we tested for changes of functional connectivity with the rest of the brain when the emotional object was a to-be-ignored distractor compared to when it was the search target [(negS_neuT + posS_neuT) > (negS_negT + posS_posT)], expecting an increased connectivity with the dorsal frontoparietal control network. Moreover, we wanted to be sure to highlight the selective functional coupling of our seed regions within the areas involved with resistance from negative, but not positive, distraction. For this reason, we used a procedure involving two masks that aimed to isolate the brain regions recruited during the avoidance of either negative or positive emotional distractors (i.e., (negS_neuT > negS_negT) and (posS_neuT > posS_posT), respectively, at *p*-*unc* = 0.05). Specifically, we used the constraint that the inter-regional coupling had to include regions recruited when coping with negative distractor (i.e., inclusive masking with negS_neuT > negS_negT), and—at the same time—not include areas involved in coping with positive distractors (i.e., exclusive masking with posS_neuT > posS_posT). This would indicate a coupling with regions selectively involved in coping with distraction by negative stimuli. An analogous masking procedure was used to assess inter-regional coupling with regions selectively recruited by positive distractors.

The statistical threshold was set to *p* = 0.05, FWE corrected at the voxel level, considering the whole brain as the volume of interest.

#### Localizer task

Together with the main search task, we acquired fMRI data during a standard localizer task for emotional-related processing areas (Johnston et al. 2010). The localizer provided us with an independent dataset to identify brain regions that responded differentially to negative vs. positive emotional stimuli, see also the “Emotional-related fMRI analysis” section above. For the localizer task, we selected 144 pictures (48 negative, 48 positive and 48 neutral pictures) from the International Affective Picture System (IAPS; Lang et al. 1999). IAPS are well-known stimuli able to elicit the activation of emotional brain areas such as the amygdala (Britton et al. 2006) or the insula (Wright et al. 2004), and have been pre-tested in normative samples for their valence and arousal values. The 144 selected IAPS pictures were clearly distinguishable according to their valence and arousal scores. The standardized mean valence scores (1 = unhappy, 9 = happy) were significantly lower for negative (3.14 ± 0.64) than for neutral (5.09 ± 0.56) pictures, which, in turn, were lower than for positive pictures (7.21 ± 0.53) [(*t*(94) = − 19,08, *p* < 0.001) and (*t*(94) = − 23.72, *p* < 0.001), respectively]. The mean arousal scores (1 = calm, 9 = excited) were greater for both negative (5.63 ± 0.55) and positive (4.73 ± 0.75) than for neutral (2.87 ± 0.54) pictures [(*t*(94) = 20.60, *p* < 0.001) and (*t*(94) = 13.79, *p* < 0.001), respectively]. The 144 selected IAPS pictures did not include any human but single objects belonging to the same categories of objects that we used as emotional target/distractor stimuli in the complex scenes of the main visual search task: e.g., for negative stimuli: scary and/or disgusting animals, dead animals, weapons, disgusting food, excrements; for positive stimuli: baby animals, appetizing foods, flowers, money, gold bars; for neutral stimuli: kitchen tools, work tools, household furniture, electrical outlets.

The participants were required to passively view the pictures. The 144 pictures were presented in 12 blocks, each consisting of 12 pictures belonging to the same emotional valence, resulting in 4 blocks of positive, 4 blocks of negative, and 4 blocks of neutral pictures. Each block lasted for 18 s (1.5 s per picture) and was separated from the following block by a variable inter-trial interval ranging from 2 to 4 s (uniformly distributed), filled with a fixation cross displayed on a gray background. To avoid the induction of long-lasting mood states, we presented the different blocks in a pseudo-random sequence so that no more than two blocks of the same condition was consecutively presented (cf. Dolcos et al. 2004).

The analysis of the functional localizer aimed to highlight the activity in limbic and para-limbic areas associated with the processing of negative valence stimuli. Accordingly, we compared “negative” minus “positive and neutral” blocks of IAPS pictures (cf. Johnston et al. 2010). This comparison revealed the activation of both the left and the right amygdala (see Table 2). As before, the statistical threshold was set to *p* = 0.05, FWE corrected at the voxel level, considering the whole brain as the volume of interest. The left and right amygdala were used as additional volumes of interest (SVC analysis) to compare scenes including negative (negs_negT and negS_neuT) vs. the other objects (neuS_neuT, posS_posT and posS_neuT) in the main visual search task, with the aim to identify regions involved in the processing of negative stimuli during the main searching task, cf. above.

## Results

### Behavioral data

On each trial, the participants indicated the location (left vs. right hemifield) of the target-object defined by the cue word. Performance was measured in terms of the “inverse efficiency score” (IES), which combines reaction times (RT) and accuracy (IES = mean RT/mean proportion of accuracy, see Table 1), and provides correction for potential speed–accuracy trade-offs present in the data (see, e.g., Bruyer and Brysbaert 2011).

**Table 1:** Behavioral data in the five experimental conditions

	NegS_negT	NegS_neuT	NeuS_neuT	PosS_posT	PosS_neuT
IES (ms)	1206 ± 43	1305 ± 51	1236 ± 38	1028 ± 26	1262 ± 32
RT (ms)	1072 ± 27	1092 ± 28	1084 ± 24	967 ± 23	1090 ± 20
ACC (%)	89.7 ± 1.5	84.8 ± 1.8	88.5 ± 1.7	94.2 ± 0.9	87.0 ± 1.8

Mean (± SEs) inverse efficiency scores (IES), reaction times (RTs), and accuracy (percentages), for the different conditions

A one-way analysis of variance (ANOVA) with five levels (i.e., the five conditions: negS_negT, negS_neuT, neuS_neuT, posS_posT, posS_neuT) was conducted on the IES data revealing significant differences among the conditions, [*F*(4, 84) = 19.31, *p *< 0.001], see Fig. 1c. Planned comparisons revealed a significant facilitation of location discrimination (lower IES) when the current to-be-searched target was an emotional object (negS_negT or posS_posT) compared to when the current target was a neutral object (negS_neuT or posS_neuT): *t*(21) = 3.31; *p *= 0.002 and *t*(21) = 8.89; *p *< 0.001, respectively (compare bars 1 vs. 2 and bars 4 vs. 5 in Fig. 1c). These findings could indicate a facilitation in selecting emotional targets (Beh H 1), but also a discrimination cost when searching for a neutral target in the presence of emotional distractors (Beh H 2).

To disentangle the discrimination facilitation vs. discrimination cost of the emotional objects depending on their current task relevance, we compared each condition including an emotional object (i.e., negS_negT, negS_neuT, posS_posT, posS_neuT) with the baseline condition, i.e., scenes without emotional objects (neuS_neuT). These planned comparisons revealed a discrimination facilitation (Beh H 1) when searching for positive targets (posS_posT; *t*(21) = 7.78; p < 0.001; compare bars 4 vs. 3 in Fig. 1c), and a discrimination cost (Beh H 2) when searching for a neutral target in the presence of negative distractors (negS_neuT; *t*(21) = 1.82; *p* = 0.041; compare bars 2 vs. 3 in Fig. 1c). By contrast, searching for a neutral target in scenes including a positive emotional distractor did not differ significantly from the baseline condition (posS_neuT; *t*(21) = 0.89; *p *= 0.192; compare bars 5 vs. 3 in Fig. 1c). This might indicate that non-target positive objects are less “distracting” than non-target negative objects compared to the baseline condition, which is in line with the literature (see Iordan and Dolcos 2015). Finally, planned comparisons failed to reveal any difference between the baseline condition and searching for negative targets (negS_negT; *t*(21) = 0.78; *p* = 0.221; compare bars 1 vs. 3 in Fig. 1c). This latter finding appears somewhat surprising with respect to the previous literature that highlighted greater discrimination or detection facilitation for negative compared to neutral objects (Flykt 2005; Öhman et al. 2001; Schubö et al. 2006)^3^. The following eye-movement data analyses were helpful to clarify this point.

### Eye-movement data

To clarify the behavioral findings (Fig. 1c and the paragraph above) and to further investigate the deployment of overt attentional resources triggered by emotional stimuli according to their task relevance, we measured three indexes related to: the latency of the first fixation on the target (f-fix-lat); the time interval between the onset of the first fixation on the target and the response button press (f-fix-RT); the duration of the first fixation on the target (f-fix-dur) (see also eye-movement analysis in the Online Resource for the target fixation probability index). We conducted three different repeated-measures ANOVAs with five levels corresponding to the main experimental conditions (negS_negT, negS_neuT, neuS_neuT, posS_posT, posS_neuT) on the data derived from each of the three fixation indexes (f-fix-lat, f-fix-RT, and f-fix-dur; see Fig. 1d). All the three ANOVAs revealed significant differences between the main conditions: f-fix-lat, [*F*(4, 84) = 2.58, *p* = 0.043], f-fix-RT, [*F*(4, 84) = 6.09, *p* < 0.001], and f-fix-dur, [*F*(4, 84) = 6.92, *p* < 0.001], indicating different patterns of fixations depending on the five experimental conditions.

Post-hoc analyses revealed that subjects’ gaze arrived equally fast on the emotional object when it was the current to-be-searched target, irrespective of being negative (negS_negT; 472 ms) or positive (posS_posT; 488 ms) (difference = 16 ms; *p* = 0.604; compare the first vs. the forth orange bar in Fig. 1d, left panel). However, RTs computed from the onset of the first fixation on the emotional target were significantly longer for negative (655 ms) than positive (542 ms) to-be-searched targets (difference = 113 ms; *p* < 0.001; compare the first vs. the forth green bar in Fig. 1d). This latter finding might account for the lack of differences in the IES performance between searching for negative targets and neutral targets in the baseline condition (see the paragraph above). Consistently, the duration of the first fixation tended to be longer for negative (753 ms) than for positive (699 ms) targets, though this effect was not fully significant (difference = 54 ms; *p* = 0.116). Overall, these patterns of eye-movement data highlighted that negative stimuli captured overt attentional orienting equally well as positive objects, supporting the EM H 1, but this did not correspond to an equally fast target-location discrimination (left vs. right response).

The analysis of the eye-movement data also revealed that the emotional objects failed to capture overt attention when task irrelevant, thus highlighting efficient top-down control to filter out current distractors (supporting the EM H 3; see also the EM H 2 for the opposite expectation). This was evidenced by the analysis of fixation indexes that directly compared emotional targets vs. emotional distractors (see Fig. 1d, right panel). We carried out two 2 × 2 repeated-measures ANOVAs with the factors of target valence (negative vs. positive) and task relevancy (emotional target vs. emotional distractor) on either the first fixation latencies (f-fix-lat) and the duration of the first fixation (f-fix-dur) data (see also the Online Resource for a consistent analysis on the fixation probability data). Both ANOVAs revealed a main effect of task relevancy [f-fix-lat: (*F*(1, 21) = 118.35, *p* < 0.001), and f-fix-dur: (*F*(1, 21) = 60.72, *p* < 0.001)], indicating that emotional distractors (i.e., negS_neuT_negF and posS_neuT_posF) were fixated later and for a shorter duration (733 ms and 414 ms, respectively) than emotional targets (i.e., negS_negT_negF and posS_posT_posF; 480 ms and 726 ms, respectively). Moreover, the ANOVA on the f-fix-dur revealed a main effect of target valence, [*F*(1, 21) = 4.47, *p* = 0.047], indicating that negative objects were fixated for a longer duration (591 ms) than positive objects (549 ms), irrespective of being targets or distractors. The ANOVAs did not reveal any other significant effects (all Fs < 2.12; all *p*s > 0.160).

Overall, these findings highlight an interplay between bottom-up and top-down attention control depending on the task relevance of the emotional stimuli. On one hand, emotional objects captured overt orienting when they were task relevant, with faster fixation latencies than non-emotional targets, though with longer RTs from target fixation (i.e., the f-fix-RT index) for negative than for positive target-objects, indicating bottom-up facilitation in directing attention towards task-relevant emotional stimuli. On the other hand, the capability of emotional objects to capture overt orienting of spatial attention was dramatically reduced when they were task irrelevant, irrespective of their positive or negative valence. This indicates efficient top-down control in filtering out the current emotional distractor. These mechanisms were further investigated through the analysis of the fMRI data.

### fMRI data

#### Target-related fMRI analysis

**Fig. 2:**
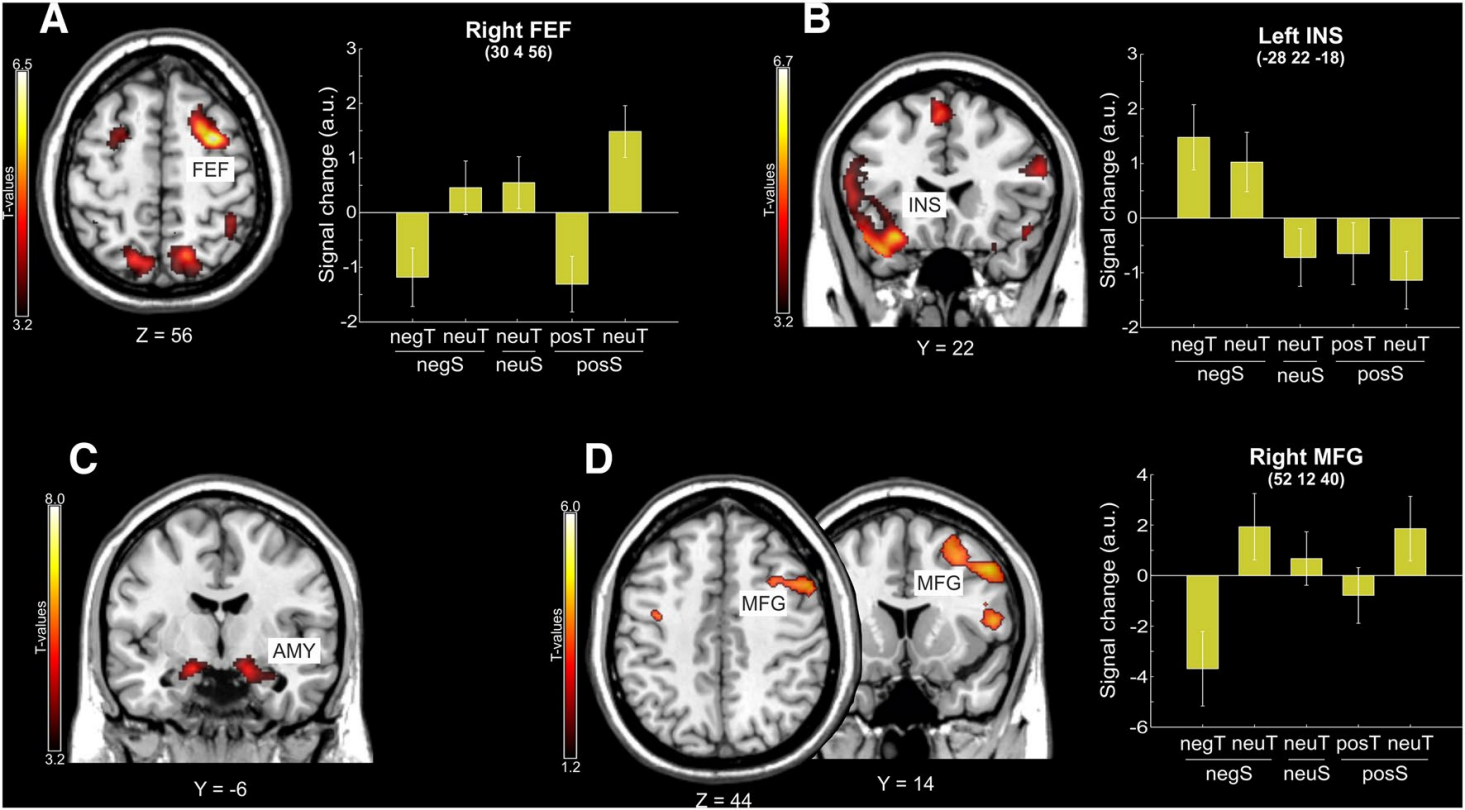
**a** fMRI results related to searching for a neutral target (neuT) in positive/negative scenes. Axial section of a standard MNI template showing the peak of activation in the right FEF in the whole brain analysis that showed a significant effect of searching for neutral vs. emotional targets irrespective of the emotional valence of the scene [(negS_neuT + posS_neuT) > (negS_negT + posS_posT)]. The bar plot summarizes the activity of the right FEF in the five conditions, highlighting an increase of activity during searching for neutral targets in emotional scenes (compare bars 2 and 5 vs. bars 1 and 4). **b** Brain responses associated with the effect of searching for negative vs. positive or neutral targets [(negS_negT + negS_neuT) > (neuS_neuT + posS_posT + posS_neuT)] at the whole brain level. The related bar plot indicates an increase of activity of the left INS during searching through negative scenes, irrespective of the task relevance of the negative object (compare bars 1 and 2 vs. bars 3, 4 and 5). **c** Coronal section showing the peak of activation in the right amygdala (AMY) that showed a significant effect of processing negative vs. positive or neutral IAPS stimuli in the localizer task [(neg) > (pos + neu)]. **d** Axial and coronal sections of a standard MNI template showing the peak of activation in right prefrontal cortex (i.e., the MFG) that showed functional coupling (psychophysiological interactions) with left INS during search of neutral vs. emotional targets [(negS_neuT + posS_neuT) > (negS_negT + posS_posT)]. The bar plot summarizes the functional connectivity of the right MFG with left INS in the five conditions, highlighting increased insular/prefrontal coupling specifically when neutral targets have to be searched for in the context of negative scenes (compare bars 2 and 5 vs. bars 1 and 4). For display purposes, all activation maps are displayed at a threshold of *p* < 0.001 (uncorrected). In the signal plot, the level of activation is expressed in arbitrary units (a.u., ± 90% confidence interval)

First, we tested for activation associated with the effect of searching for “emotional minus neutral targets” in the emotional scenes, i.e., [(negS_negT + posS_posT) > (negS_neuT + posS_neuT)], expecting to observe increased activity in limbic/para-limbic regions, given the bottom-up facilitation in directing attention towards task-relevant emotional stimuli (see fMRI H 1). However, this comparison failed to reveal any significant effect at the whole brain level, possibly due to the fact that we averaged negative and positive stimuli (see also Introduction). Conversely, the opposite contrast, related to searching for neutral targets in the presence of emotional distractors, irrespective of their emotional valence, [(negS_neuT + posS_neuT) > (negS_negT + posS_posT)], highlighted activity in a network of dorsal frontoparietal regions typically involved with top-down control (Corbetta and Shulman 2002; Corbetta et al. 2008; see also Iordan et al. 2013), supporting the fMRI H 2. These regions included anteriorly the right frontal eye fields (FEF), and posteriorly, the right superior parietal gyrus (SPG), plus several activations in the occipital cortex, including the right and left lingual gyri (LG) and the right middle occipital gyrus (MOG; see Table 2). Although this network of areas was more pronounced on the right hemisphere, homologous activations were also observed at a lower statistical threshold in the left hemisphere (see Fig. 2a).

**Table 2:** MNI coordinates (*x*, *y*, *z*), *Z* values, and *p* values for the areas showing a significant activation in the main visual search task and in the localizer task

	*x y z*	*Z* value	p-FWE-corr
Searching for neutral targets in emotional scenes			
Right FEF	30 4 56	5.81	< 0.001
Right SPG	16 − 64 58	4.88	0.016
Right LG	10 − 98 12	5.24	0.003
Left LG	− 8 − 100 16	5.24	0.007
Right MOG	36 − 82 36	5.02	0.008
Searching for negative vs. positive plus neutral targets			
Left IFG	− 52 36 6	6.03	< 0.001
Right IFG	50 34 6	5.97	< 0.001
Left INS	− 28 22 − 18	5.26	0.003
MSFc	− 4 28 50	5.22	0.003
Left STP	− 46 24 − 18	5.03	0.008
Left FG	− 46 − 52 − 22	5.52	0.001
Left IOG	− 46 − 62 − 14	5.40	0.001
Left MOG	− 50 − 76 4	5.22	0.003
Left MTG	− 58 − 66 0	4.89	0.019
Right IOG/ITG	44 − 72 − 8	6.01	< 0.001
Processing of negative vs. positive plus neutral IAPS pictures in the localizer task			
L AMY	− 16 − 6 − 16	4.82	0.011
R AMY	16 − 6 − 16	4.64	0.022
L MOG	− 48 − 80 8	6.64	< 0.001
R MOG	48 − 76 18	5.70	< 0.001
L SOG	− 26 − 82 30	5.16	0.002
L FG	− 44 − 58 − 18	4.44	0.050
R FG	44 − 50 − 26	5.49	< 0.001
L LG	− 22 − 76 − 10	4.89	0.008
SMA	10 16 70	5.17	0.002
R IFG	42 8 26	4.68	0.019

*FEF* frontal eye fields, *SPG* superior parietal gyrus, *LG* lingual gyrus, *MOG/SOG/IOG* middle/superior/inferior occipital gyrus, *IFG* inferior frontal gyrus, *INS* insula, *MSFc* medial superior frontal cortex, *STP* superior temporal lobe, *FG* fusiform gyrus, *MTG* middle temporal gyrus, *ITG* inferior temporal gyrus, *AMY* amygdala, *SMA* supplementary motor area

Considering the searching cost revealed by our behavioral analysis with negative distractors, we checked whether the activity of the dorsal frontoparietal network was specifically related to the avoidance of negative vs. positive distractors [(negS_neuT—negS_negT) > (posS_neuT—posS_posT)] or vice versa [(posS_neuT—posS_posT) > (negS_neuT—negS_negT)]. Both analyses failed to reveal any significant activations, suggesting that both negative and positive emotional distractors contributed in the enhanced activity observed in the dorsal frontoparietal network during avoidance of emotional distraction.

#### Emotional-related fMRI analysis

We then focused on the processing of emotional valence. First, we identified areas responding to negative stimuli by comparing the two conditions including a negative object, irrespectively of their task relevance (negS_negT and negS_neuT) vs. the other three conditions (see fMRI H 3). At the whole brain level, this revealed a large network of areas, including anteriorly the left and right IFG, the left insula (INS) and the medial superior frontal cortex (MSFc) (Fig. 2b). Significant effects were also found in several occipital and temporal areas, including the left FG, the left MOG, the left and right inferior occipital (IOG) and temporal (ITG) gyri (see Table 2). The opposite comparison including positive objects irrespectively of their task relevance (posS_posT and posS_neuT) vs. the other three conditions failed to reveal any significant activations, suggesting a selective involvement of the above described regions during the processing of negative (but not positive) stimuli, irrespective of their current task relevancy.

In addition, we used the localizer data to highlight areas traditionally associated with the processing of negative stimuli (cf. Table 2, Fig. 2c, and method section). The comparison between “negative” vs. “positive and neutral” IAPS pictures administered with the localizer task revealed the activation of the left (x, y, z: − 16, − 6, − 16; *t* = 4.82; *p* = 0.011) and right amygdala (x, y, z: 16, − 6, − 16; *t* = 4.64; *p* = 0.022). We then used the coordinates of these areas as additional regions of interest to search for the effect of processing negative stimuli [“scenes including a negative object (negs_negT and negS_neuT)” minus “the other three conditions (neuS_neuT, posS_posT and posS_neuT)”] in the main visual search task. This revealed a significant effect of processing negative scenes in the right amygdala (x, y, z: 18, − 4, − 20; *t* = 3.25; *p*-*SVC*-*corr* = 0.023), but not in the left amygdala (x, y, z: − 20, − 6, − 18; *t* = 2.33; *p*-*SVC*-*corr* = 0.152). The activation of the right amygdala confirmed the fMRI H 3, expecting stronger involvement of the limbic system during the processing of the negative scenes, irrespective of the task relevance of the negative objects. Again, no effects were found for the processing of positive stimuli.

Limbic/para-limbic areas involved in the processing of scenes including a negative object (namely the left insula and the right amygdala) were combined together in a volume of interest (VOI) to test whether their activity was modulated by task relevance. For this, we contrasted, within this VOI, scenes where the negative object was task relevant vs. scenes where the negative object was task irrelevant [(negS_negT) > (negS_neuT)] and vice versa [(negS_neuT) > (negS_negT)]. These analyses failed to reveal any significant activation, apparently indicating that the activity in these limbic/para-limbic areas was not further modulated by the current task relevance (i.e., by top-down control).

#### Inter-regional connectivity of the affective regions

The evidence that emotional objects capture overt attention to a less extent when they are task irrelevant (cf. eye-movement data; see also Fig. 1d, right panel) is consistent with the notion of a selective involvement of top-down control to cope with emotional distractors. Based on this evidence and on the literature highlighting changes of connectivity between limbic/para-limbic regions and other cortical regions involved in attention control during coping with emotional distraction (cf. Dolcos et al. 2006; Iordan et al. 2013; Uddin 2015; see also Introduction), we chose the left insula and the right amygdala (i.e., the regions responding to negative scenes in the emotion-related fMRI analysis, cf. Table 2) as regions of interest (ROI) for the PPI analysis. For each of the two seed regions, we tested for changes of functional connectivity with the rest of the brain when the emotional object was a to-be-ignored distractor compared when the emotional object was the search target [(negS_neuT + posS_neuT) > (negS_negT + posS_posT)], expecting increased coupling between limbic/para-limbic and dorsal top-down control regions in the presence of emotional distractors (see fMRI H 4).

When the connectivity analysis was seeded on the right amygdala, we failed to observe any significant functional coupling with the rest of the brain. Instead, the left insula showed significant connectivity with the right prefrontal cortex (*x*, *y*, *z*: 52, 12, 40; *t *= 5.18; *p* = 0.022), extending dorsally with the FEF and ventro-laterally with the MFG (see Fig. 2d). Since the seed regions for the PPI analysis (i.e., left insula and right amygdala) derived from the comparison between “negative scenes” minus “neutral and positive scenes”, we aimed to specifically highlight the functional connectivity of these regions in the presence of negative distractors. For this reason, we used a masking procedure (see Methods), confirming that the inter-regional coupling between the left insula and the right prefrontal cortex was present inside the neural circuit recruited when coping with negative distractor (i.e., inclusive masking with negS_neuT > negS_negT, *p*-*unc*. = 0.05). On the contrary, it was absent inside the neural circuit recruited when coping with positive distractor (i.e., inclusive masking with posS_neuT > posS_posT, *p*-*unc*. = 0.05). This masking procedure, therefore, allowed to highlight the selective coupling between the insular and prefrontal cortex when coping with negative but not positive distraction. The signal plot of Fig. 2d highlights the specificity of this effect in the context of coping with negative distraction: insular/prefrontal coupling increased when searching for neutral targets with negative distractors and decreased when searching for negative targets (compare bars 2 vs. 1), while positive scenes modulated much less the functional coupling between these areas (compare bars 5 vs. 4). Ultimately, these findings highlighted an enhanced insular/prefrontal coupling selectively when neutral vs. emotional targets have to be searched for while coping with distraction from negative elements in the scene, confirming the fMRI H 4.

#### Discussion

The aim of the current study was to investigate at both behavioral and neural levels the impact of emotional stimuli on the distribution of attentional resources depending on their current task relevance, i.e., emotional targets vs. emotional distractors. The current behavioral findings revealed overall facilitation in searching for emotional compared to neutral targets in the presence of emotional distractors. Surprisingly, this benefit was selective for positive stimuli. Nevertheless, the analysis of fixations revealed that both negative and positive targets were fixated earlier than neutral targets, indicating that they captured overt spatial attention to a comparable extent. Notwithstanding that, attentional capture by emotional stimuli was modulated by the current task demand: emotional distractors were fixated later and for a shorter duration than emotional targets, suggesting efficient top-down control in avoiding emotional distraction, at least in terms of the overt distribution of spatial attention, while negative distractors still entailed a searching cost in terms of behavioral performance. Consistently, the fMRI data demonstrated an interplay between bottom-up and top-down processes to cope with emotional distraction. Negative (but not positive) stimuli were mandatorily processed by limbic/para-limbic regions (namely the left insula and the right amygdala) irrespective of the current task relevancy. As revealed by the analysis of inter-regional connectivity, however, the functional coupling between the left insula and the right prefrontal cortex increased while searching for neutral targets, specifically in the presence of negative emotional distractors. This indicates that the inter-regional coupling between affective and attention control regions plays a central role to attenuate emotional distraction.

The behavioral facilitation in searching for emotional compared to neutral targets is in agreement with most of the previous literature, indicating a global facilitation in processing emotional stimuli (Engen et al. 2015; Pedale et al. 2017; Schupp et al. 2003), as well as in searching for emotional vs. neutral targets in displays including an emotional distractor (Flykt 2005; Öhman et al. 2001). Compared with searching for neutral targets in neutral scene, however, only positive (but not negative) targets entailed faster location discrimination, which might appear somewhat surprising. The large majority of emotional studies employed negative stimuli, while only a few studies have focused on the effect of positive emotional stimuli (e.g., Anderson et al. 2011; Bradley et al. 2004; van Hooff et al. 2011). The general finding is that positive stimuli associated with rewards (e.g., sexual stimuli, drugs-related stimuli for addicted individuals, or stimuli associated with reward via conditioning) tend to capture attentional resources equally well than negative stimuli. Here, we showed an advantage in searching for positive stimuli even though they were not associated with any specific reward. A similar finding was observed by Hodsoll and colleagues (2011). They used a visual search task in which participants were asked to search for a singleton target face among distracting faces (i.e., a female face among male faces or vice versa) and then judge the target face orientation (see also the description of this study in the Introduction). When one of the distractor faces had an emotional expression (fearful, angry or happy), the target face orientation discrimination was impaired. However, when the target face was an emotion singleton, only happy faces involved RT facilitation, indicating attentional capturing. Hodsoll and colleagues explained this finding suggesting “the possibility that the cost associated with processing negative expressions is not merely due to capture of attention to the wrong item (distractor rather than target). There appears to be an additional effect of slowing simply due to the very processing of the negative emotion and its unpleasant connotations. Such cost can offset any potential benefit of capture to negative target singletons and may result in a difficulty to disengage from negative emotional faces” (p. 352).

Here, we reported consistent findings with Hodsoll and colleagues (2011), with search benefits following positive stimuli, and search costs following negative stimuli (cf. Fig. 1c). Crucially, the current data on fixation patterns allow us to confirm and further extend the notion of “negative-related disengagement difficulty” to objects other than faces in complex visual scenes. The current eye-movement data corroborate this view, highlighting that negative stimuli were equally attentional grabbing than positive stimuli when task relevant. This latter finding was evidenced by similar indexes of first fixation latencies (i.e., the f-fix index; see also the fixation probability index on the Online Resource) on both positive and negative emotional targets (cf. Fig. 1d). However, the mean RT measured since the first fixation on target revealed disproportionately longer response latencies (i.e., the f-fix-RT index) for negative than for positive target-objects. This indicates that, although attentional grabbing, the negative stimuli entailed slower responses. This may arise from a difficulty to disengage from processing the negative emotional attributes of the object (cf. Hodsoll et al. 2011). The analysis of fixation patterns also revealed that the strength of (overt) attentional capture by emotional stimuli is modulated by the current task relevance. In fact, first fixation latencies on emotional distractors were about 300 ms slower than first fixations on emotional targets, indicating efficient avoidance of emotional distraction (cf. Fig. 1d). In summary, these results supported a twofold effect driven by emotional objects: on one hand, a facilitation in directing the gaze to the emotional objects when they were the current targets, indicating the existence of attentional priorities related to these stimuli; on the other hand, subjects’ gaze avoided the emotional stimuli when they were task irrelevant, indicating efficient top-down control to cope with emotional distractors.

The efficient top-down attentional control to avoid overt orienting of spatial attention on emotional distractors is consistent with our fMRI data. Searching for neutral targets in scenes including emotional distractors revealed the recruitment of the dorsal frontoparietal network, typically involved with top-down volitional control (Corbetta and Shulman 2002; Corbetta et al. 2008). Dorsal frontoparietal regions, including the superior premotor cortex and the posterior parietal cortex, have been shown to be consistently involved during active visual search and target detection using naturalistic stimuli (e.g., Ellison et al. 2014; Ogawa and Macaluso 2015). More generally, these regions have been shown to be involved with attention control and voluntary shifts of spatial attention (e.g., Corbetta and Shulman 2002; Corbetta et al. 2008; Hahn et al. 2006). The increased activation of these regions when participants need to avoid emotional distractors to carry out the searching task is in good agreement with the current eye-movement results, indicating efficient filtering out of task-irrelevant emotional stimuli. Both findings suggest an increased necessity of top-down control to resist from directing the gaze and processing resources to emotional distractors.

Notwithstanding the recruitment of the top-down frontoparietal control system, the impact of negative stimuli on searching performance is evident when looking at the searching costs, with a decreased performance in searching for neutral targets in the presence of negative (but not positive) distractors compared to searching for neutral targets in neutral scenes (cf. bar 2 vs. bar 3 in Fig. 1c). This latter finding suggests that negative stimuli did capture attention resources, at least in a “covert” fashion (cf. first fixation latencies on emotional distractors about 300 ms longer than on emotional targets), with a consequent detriment of searching performance due to negative emotional distraction (e.g., Anticevic et al. 2010; Dolcos et al. 2006). The behavioral effect of negative distraction is also consistent with the evidence that negative stimuli here activated limbic/para-limbic regions, irrespective of their being relevant or not for the search task (see the left insula, Fig. 2b, and the right amygdala). The involvement of these regions in the processing of negative stimuli has been demonstrated by a number of previous studies (see, for reviews, Fox et al. 2015; Lindquist et al. 2012; Uddin 2015). Here, we extend these results showing increased limbic/para-limbic activity during the exploration of complex visual scenes, including a number of non-emotional objects competing for processing resources with the emotional object.

These findings in limbic/para-limbic regions support the notion of a “mandatory” processing of negative emotional stimuli, irrespective of current task demands. For instance, Vuilleumier and colleagues (2001) asked participants to judge similar vs. different pairs of faces or houses presented in attended or unattended locations. The presentation of fearful faces reflected in increased activity of the amygdala, irrespective of any manipulation of spatial attention (though see Pessoa et al. 2002, for inconsistent findings). Similarly, in the context of visual search, Fenker and colleagues (2010) reported a magnetoencephalography (MEG) study in which they manipulated the difficulty (high vs. low) of searching for stimuli superimposed on task-irrelevant fearful vs. neutral faces. Task-irrelevant fearful faces elicited the activation of the extra striate visual cortex, irrespective of high or low searching demands and searching performance. Consistently, here we found increased limbic/para-limbic activity (namely in the left insula and in the right amygdala) whenever a negative stimulus occurred in the scene, which, therefore, appears to be unavoidably processed in a mandatory fashion.

The current task demand nevertheless modulated the functional connectivity of these regions, and, in particular, of the left insula. This para-limbic region has been recently proposed to act as a central “hub” integrating both internal information, involving bodily state and other signals from the adjacent limbic regions, and external information related to emotionally salient stimuli, thus coordinating the activity of control/monitor structures such as the anterior cingulated cortex and prefrontal regions along the dorsal frontoparietal cortex (Menon and Uddin 2010; Uddin 2015). The key role of the insular cortex as a central “emotional” hub has been supported by previous functional connectivity studies (e.g., Cauda et al. 2011; Nomi et al. 2016; Taylor et al. 2009; see Uddin et al. 2014, for a review). Here, we directly demonstrate using an active task (i.e., a visual search) the crucial role of this region in processing emotional saliency and in coordinating top-down (dorsal frontoparietal) resources when the emotional stimulus is task irrelevant. The enhanced insular/prefrontal connectivity in conditions of emotional distraction could reflect the engagement of a neural circuit in which the insula plays first the role of “emotional salience detector” (Cauda et al. 2011; Seeley et al. 2007; Uddin 2015). Then, to cope with emotional interference/distraction, the insula would determine a switch from exogenous to endogenous attentional control, through the increased connectivity with the dorsal frontoparietal control network (Bishop et al. 2004; Vincent et al. 2008). In line with this notion, here we found an increased insular activation in the presence of negative scenes, irrespective of the current relevance of the emotional object, but also a selective increase in the insular/prefrontal connectivity in conditions of searching for neutral targets in the presence of negative distractors (cf. Fig. 2d). It is worth noting that this condition (negS_neuT) entailed a behavioral cost compared to the baseline condition (neuS_neuT; i.e., searching for a neutral target without any emotional distractor), suggestive of the recruitment of additional resources (top-down control) to cope with emotional distraction and carry out the search task. The increased insular/prefrontal coupling can be then interpreted as representing, on one hand, the involvement of a mandatory processing of negative stimuli accomplished by the insula despite task irrelevance, and, on the other hand, the attempt to attenuate emotional distraction by the recruitment of neural top-down control resources to perform the visual search task. The latter would include the right MFG extending dorsally to the FEF, activated by searching for neutral (non-emotional) targets (cf. Fig. 2a).

To conclude, the current findings highlighted an interplay between bottom-up attention driven by emotionally salient negative stimuli, processed by ventral affective areas (namely insula and amygdala) and top-down control, processed by dorsal executive/control regions, during search for neutral targets in the presence of emotional distractors. Here, we demonstrated that the increased functional coupling between affective and control regions (i.e., the insular/prefrontal connectivity) is a core mechanism enabling the avoidance of emotionally negative distractors, thus allowing the deployment of spatial attention resources toward task-relevant emotionally neutral stimuli during visual search in complex and naturalistic scenes.

## Electronic supplementary material

Below is the link to the electronic supplementary material.

Fig. S1 Supplementary Eye-movement analysis. Mean ± standard error of the fixation probability index related to fixations of the current target-object in the main conditions (negS_negT_negF, negS_neuT_neuF, neuS_neuT_neuF, posS_posT_posF, posS_neuT_neuF) or to fixations of the emotional object when it was not the to-be-searched target in the negS_neuT_negF and posS_neuT_posF conditions.

Fig. S2 Projections on a standard MNI template showing activation related to the processing “negative minus positive” scenes (red map), “negative minus neutral” scenes (yellow map), and the overlap between the two comparisons (orange map). Activation maps are displayed at a threshold of p < 0.001 (uncorrected).

Supplementary material 1 (PDF 324 kb)

## Footnotes

1 It is worth noting that, despite our set of stimuli included emotional stimuli clearly distinguishable from the neutral stimuli in terms of valence and arousal, the absolute difference in valence and in arousal ratings between neutral and positive scenes was lower than the difference between neutral and negative scenes. This potential limitation is a consequence of the fact that we used a limited set of emotional categories. Stimuli eliciting highly arousing positive emotions typically represent social interactions (e.g., families, sexual stimuli, romantic couples; see the IAPS database, Lang et al. 1999). Conversely, the scenes used for the current task did not include any human subject, which might result to be more salient than other objects, and then, attentional capturing per se.

2 Note that for the two latter conditions in which the emotional object was a distractor we did not compute the f-fix-RT index: given that here we analyzed fixations related to task-irrelevant objects that did not require any responses, it was pointless to report for these conditions the interval between the first fixation and response button press (i.e., the f-fix-RT index), which is related to a different object in the scene (i.e., the current to-be-searched neutral target).

3 For completeness, we conducted two one-way ANOVAs on the main conditions (negS_negT, negS_neuT, neuS_neuT, posS_posT, posS_neuT) for the accuracy and RT data. These revealed overall coherent results with the IES. Both analyses were significant: ACC [*F*(4, 84) = 6.16, *p* < 0.001] and RTs [*F*(4, 84) = 21.5, *p *< 0.001]. Planned comparisons revealed higher accuracy when the current target was an emotional (negS_negT or posS_posT) compared to a neutral object (negS_neuT or posS_neuT): *t*(21) = 2.75; *p* = 0.006 and *t*(21) = 3.76; *p *< 0.001, respectively. Moreover, planned comparisons revealed faster RT for positive targets (posS_posT) compared to neutral targets in the presence of positive distraction (posS_neuT): *t*(21) = 10.17; *p *< 0.001. As concerns the baseline condition (neuS_neuT), the planned comparisons revealed faster RT and higher accuracy when searching for positive targets (posS_posT): *t*(21) = 7.43; *p* < 0.001 and *t*(21) = 3.36; *p* = 0.001, respectively; and only higher accuracy when searching for negative targets (negS_negT): *t*(21) = 1.77; *p* = 0.046.
