# Supplementary material for: Enhanced insular/prefrontal connectivity when resisting from emotional distraction during visual search
Source: Brain Struct Funct. 2019 May 21;224(6):2009–26. doi: 10.1007/s00429-019-01873-1 (PMC6591190; doi:10.1007/s00429-019-01873-1)
Supplement: Supplementary file 1 — Supplementary material 1 (PDF 324 kb) [file 429_2019_1873_MOESM1_ESM.pdf]

Enhanced insular-prefrontal connectivity

when resisting from emotional distraction during visual search

Tiziana Pedale, Emiliano Macaluso & Valerio Santangelo

Full-Length Paper to Brain Structure and Function

Corresponding Author:
Valerio Santangelo,
Dept. of Philosophy, Social Sciences & Education
University of Perugia, 06123, Perugia (Italy)
E-mail: valerio.santangelo@unipg.it

SUPPLEMENTARY MATERIAL

Supplementary Eye-movement analysis

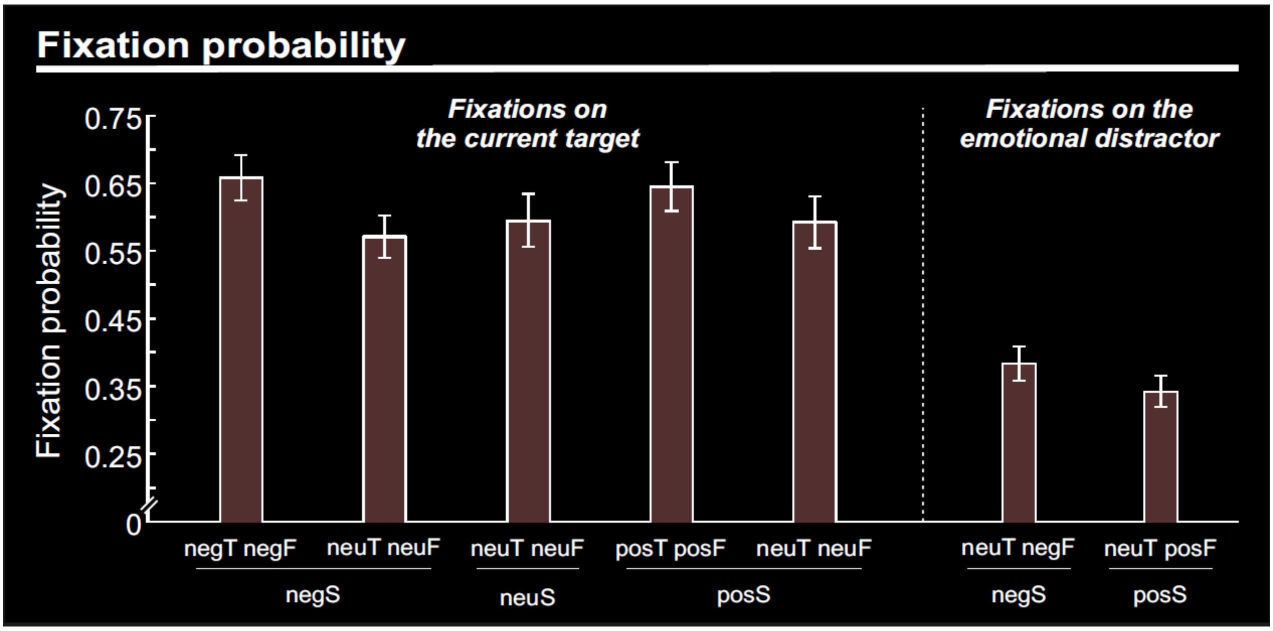

Figure S1: Mean ± standard error of the fixation probability index related to the current target-object in the main conditions (negS_negT_negF, negS_neuT_neuF, neuS_neuT_neuF, posS_posT_posF, posS_neuT_neuF) or to the emotional object when it was not the to-be-searched target in the negS_neuT_negF and posS_neuT_posF conditions.

Together with the fixations indexes reported on the main manuscript, we also measured the fixation probability of the target-map (fix-prob), that is, the average across subjects of the proportion of targets fixated at least once in that condition. As for the main fixation indexes reported on the manuscript, we first conducted a repeated-measures ANOVA with five levels corresponding to the main experimental conditions (negS_negT_negF, negS_neuT_neuF, neuS_neuT_neuF, posS_posT_posF, posS_neuT_neuF) on the data derived from fix-prob. The ANOVA revealed significant differences between the main conditions: [$F(4, 84) = 3.25$, $p = .016$], indicating different target fixation probabilities depending on the experimental condition. Post-hoc analyses revealed that the fixation probability of negative (0.66) and positive (0.65) task-relevant objects was similar (difference = 0.01; $p = .680$; compare bar 1 vs. 4), and higher than for the other conditions (bar 1 vs.

2: p = .004; bar 1 vs. 3: p = .033; bar 1 vs. 5: p = .029; bar 4 vs. 2: p = .013; bar 4 vs. 3: p = .082; bar 4 vs. 5: p = .074). Next, we conducted a 2x2 repeated-measures ANOVA to compare the fixation probability of emotional objects depending on their current task-relevance. This analysis included the factors of Target valence (negative vs. positive) and Target relevance (task-relevant vs. irrelevant) on fixation probability data. The ANOVA revealed a main effect of target relevance, [$F(1, 21) = 112.76$, $p < .001$], indicating that task-irrelevant emotional objects were fixated with an overall less probability (0.36) than task-relevant emotional objects (0.65). The ANOVA did not revealed any other significant effects (all Fs < 2.07; all ps > .164).

Supplementary Figure

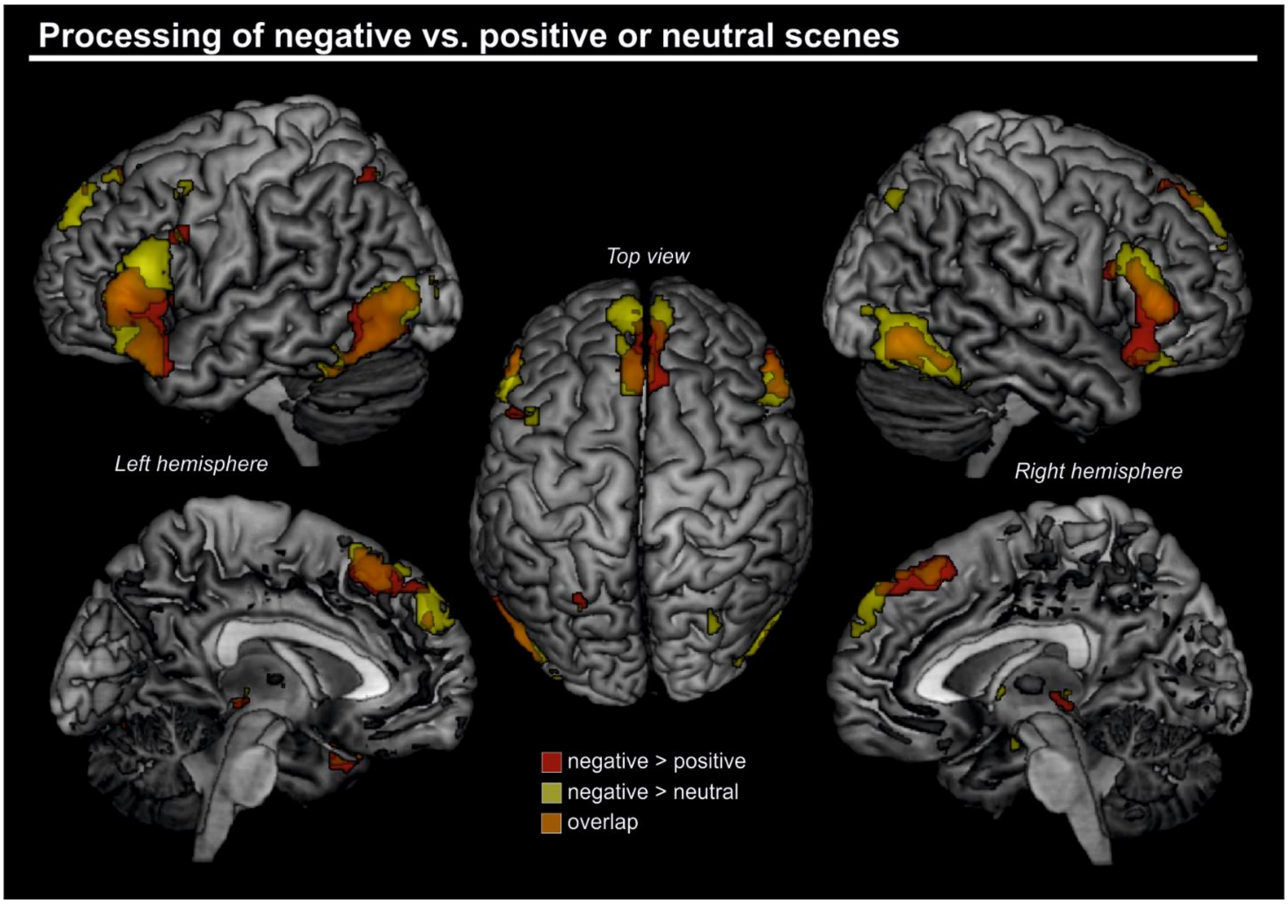

Figure S2: Projections on a standard MNI template showing activation related to the processing "negative minus positive" scenes (red map), "negative minus neutral" scenes (yellow map), and the overlap between the two comparisons (orange map). Activation maps are displayed at a threshold of $p < .001$ (uncorrected).
